# Hemorrhoids Treated with Capsicum

**Published:** 1880-07

**Authors:** 


### HEMORRHOIDS TREATED WITH CAPSICUM.

In cases of hemorrhoidal congestion, Vidal regards capsicum annum as the best remedy. He prescribes four or five pills daily, each containing 20 centigrams, half at breakfast time and half at supper time. Under this influence the congestion and all the painful symptoms which accompany it disappear rapidly —Vidal in *Journ. de Med.*, Feb. 1880, p. 70.

